# Editorial: Learning for action in policy implementation

**DOI:** 10.3389/frhs.2024.1515478

**Published:** 2024-11-19

**Authors:** Yanfang Su, Heather L. Bullock, Michael Trisolini, Karen M. Emmons

**Affiliations:** ^1^ Department of Global Health, School of Public Health, University of Washington, Seattle, WA, United States; ^2^ Evans School of Public Policy and Governance, University of Washington, Seattle, WA, United States; ^3^ Department of Health Research Methods, Evidence and Impact, McMaster University, Hamilton, ON, Canada; ^4^ Waypoint Research Institute, Waypoint Centre for Mental Health Care, Penetanguishene, ON, Canada; ^5^ Department of Public Health and Health Sciences, Northeastern University, Boston, MA, United States; ^6^ Department of Social and Behavioral Sciences, Harvard T.H. Chan School of Public Health, Boston, MA, United States

**Keywords:** learning for action, evidence, policy, implementation science, implementation strategies

**Editorial on the Research Topic**
Learning for action in policy implementation

## Introduction

Policy implementation science (IS) is an emerging field that intersects implementation science and public policy studies to support the translation of evidence into policy. As a multidisciplinary field, Policy IS uses methodologies and frameworks from economics, political science, sociology, public administration, knowledge translation, and other fields. Policy IS would benefit from broader consensus on definitions, theories, frameworks, methodologies, and outcomes, so that a wide range of studies could build on a common conceptual foundation and thus build scientific knowledge more rapidly. The objective of this Research Topic is to reach consensus and drive learning for action in policy implementation by strengthening connections among a range of policy IS working partners, including implementation scientists, policy researchers, technical advisers, policymakers, and policy implementation practitioners.

In this special collection, 82 authors contributed to publishing 12 manuscripts on topics relevant to the design, processes, and impacts of policy implementation. The collection includes one theoretical discussion on global context (List and Agamile et al.); reviews on service integration (Tao et al.) and tobacco licensing (Bera et al.); methodological studies on equity-centered hybrid policy implementation studies (Asada et al.), quantitative policy IS measures (Smith et al.) and the integration of legal epidemiology and IS (Lane and Stergachis); one measurement study on acceptability and feasibility of policy implementation strategies (Purtle et al.); one qualitative comparative case study on policy intermediaries (Bullock et al.); and one case study on food assistance policy implementation (Kenney et al.); one quantitative evaluation effective communication related to policy IS (Dodson et al.); one mixed-method study on inter-sector care for the homeless (Martins et al.); one design study on participatory development of a target policy profile (Means et al.). Reviewing the papers included in this Research Topic, we identified several themes salient to the future of policy IS.

## Working definitions of policy implementation

Policies include regulatory, promotional, and redistributive decisions and guidelines for implementing programs to achieve societal goals (1). Lane and Stergachis addressed the importance of systematic collection and coding of laws to enable policy implementation analysis. It is notable to observe that a consensus was implicitly reached in the 12 manuscripts regarding the importance of the evidence base for policy and practice (EBPP). The working definition of policy IS in the 12 manuscripts aligns with the National Cancer Institute definition of policy IS (2). However, from a policy perspective, research evidence is not the only input into policy decisions and that is why evidence-informed policies and practices (EIPP) was raised as a critical concept (3). In public administration, policy implementation is defined as a deliberate, sanctioned change to public policy legitimized by a political authority, with an emphasis on changing the status quo and adapting to diverse contexts (4). Scientific evidence is considered as one of multiple resources in implementation. Policy implementation strategies include information campaigns (Kenney et al.), licensing (Bera et al.), as well as others (e.g., contracting, subsidies, accreditation). Through literature review and synthesis, Tao et al. highlighted policy implementation strategies, including training, resource reallocation, and increased insurance coverage.

## Theories and conceptual frameworks for policy implementation

Policy IS has distinctive challenges in different settings and contexts (e.g., global vs. domestic). Incorporating contextual contingencies is important to address the factors affecting policy IS. List and Agamile et al. brought the discussion of global policy implementation in decolonizing global health. This includes the application of frameworks and processes through a global perspective that aligns with diverse governance, power, resources, stakeholder relationships and health systems. They highlighted opportunities for reimagining policy implementation science across the policy cycle from agenda setting and policy formulation, to policy implementation and evaluation using real world examples.

In synthesizing scientific evidence, conceptual frameworks are crucial to cluster the findings and aggregate knowledge, with the potential of theorization. Health Triangular Policy Framework by O'Brien et al. was applied in the narrative review by Tao et al., with an emphasis on actor-relevant contexts, contents, and processes (5). Comparatively, Bera et al. applied a framework for contextual analysis, categorizing strength, weaknesses, opportunities, and threats, in tobacco retailer licensing as a crucial policy implementation strategy in regulating tobacco access.

Several papers present examples of using policy IS frameworks to drive implementation evaluation and outcomes. For instance, Means et al., Kenney et al., and Smith et al. directly linked implemented policies to implementation outcomes (e.g., reach, adaptability) and final outcomes relevant to health and equity. Kenney et al. illustrated the utility of a policy IS framework by Bullock et al. through a case study (3). Asada et al., Bullock et al. demonstrated how frameworks from related fields of political science, public policy, and IS can inform policy IS.

## Methods for studying policy implementation

This Research Topic represented a wide range of empirical evidence from surveys (e.g., Purtle et al., Dodson et al.), comparative case studies (e.g., Bullock et al., Smith et al.), mixed-method studies (e.g., Martins et al.) and design studies (Means et al.).

In survey-based studies with policymakers and implementers, low response rates are a general challenge. For example, Dodson et al. had a 4.5% response rate in a national survey of local officials. Nonetheless, the results from these studies shed light on policy implementation practice. Dodson et al. studied the strategies to deliver policy briefs to facilitate information dissemination to local policymakers. Their study found that the narrative policy briefs had the lowest score (42%) related to strength in reasoning. In contrast, usual-care and risk-framing brief types had significantly higher scores to reflect strong reasoning (59% and 52%, respectively).

This Research Topic collected diverse studies to learn for action. Bullock et al. elaborated the role of intermediaries in implementing mental health policies through document analysis, site visits, and interviews in three distinctly different high-income countries. Smith et al. discussed four design considerations of policy implementation measures using three case studies. Martins et al. used secondary quantitative data and documents as well as collected first-hand data through interviews and focus groups to study tailored inter-sector care in the COVID-19 pandemic among the homeless. Means et al. showcased the holistic development of a Target Policy Profile as a single document to guide future work.

## Looking forward

Challenges in policy IS include the complexity of developing overarching theories that address diverse contexts and evolving implementation partners. Contextual analysis often lacks direct causal links to outcomes, while randomized trials are difficult due to the nature and scale of policy implementation. Other useful policy IS methods include legal epidemiology (Lane and Stergachis) and coincidence analysis (6). Additionally, measuring outcomes across macro, meso, and micro levels, accounting for both intended and unintended effects, remains complex.

A limitation of this Research Topic is that all of the manuscripts are in health and related domains such as food and nutrition (Kenney et al., Smith et al.) and housing instability (Martins et al.). We anticipate that future Research Topics will cover other social policies, such as unemployment, poverty, education, and LGBTQIA+ marriage. We also anticipate future studies on systematic reviews to understand the overarching landscape of policy implementation, modeling studies to predict policy impacts, costing methods for use in policy implementation, and others.
